# Baseline co-occurrence of *Faecalibacterium prausnitzii* and *Ruminococcus bromii* is associated with greater increases in selected short-chain fatty acid-related pathways following resistant starch type 2 intervention across diverse populations

**DOI:** 10.3389/fmicb.2026.1959463

**Published:** 2026-10-07

**Authors:** Yue Ma, Yu Lu, Mingyan Geng, Yan Wang, Wei-Hua Chen

**Affiliations:** 1 Shandong Medical and Pharmaceutical University, Yantai, Shandong, China; 2 Key Laboratory of Molecular Biophysics of the Ministry of Education, Hubei Key Laboratory of Bioinformatics and Molecular-imaging, Center for Artificial Intelligence Biology, Department of Bioinformatics and Systems Biology, College of Life Science and Technology, Huazhong University of Science and Technology, Wuhan, China

**Keywords:** *Bifidobacterium adolescentis*, *Faecalibacterium prausnitzii*, gut microbiome, *Ruminococcus bromii*, short-chain fatty acids, type 2 resistant starch

## Abstract

Type 2 resistant starch (RS2) can be fermented by the gut microbiota and contribute to short-chain fatty acid (SCFA) production; however, microbial responses to resistant starch vary substantially across individuals and populations, and the role of baseline microbiota in this variability remains incompletely understood. In this study, we integrated four publicly available paired metagenomic datasets representing diverse experimental and host contexts and performed cross-dataset analyses of taxonomic and functional responses. *Faecalibacterium prausnitzii* was among the taxa consistently enriched after resistant starch-related intervention or exposure across multiple datasets. Exploratory baseline stratification further suggested that the presence of *F. prausnitzii* was associated with changes in selected SCFA- and carbohydrate metabolism-related pathways. We subsequently examined its relationships with the representative resistant starch degraders *Ruminococcus bromii* and *Bifidobacterium adolescentis*. Baseline association analyses identified a reproducible positive association between *R. bromii* and *F. prausnitzii*. In cohort-adjusted analyses of mutually exclusive microbial configurations, the Fp + Rb configuration was associated with greater increases in the acetate/lactate-related pathways P41-PWY and PWY-5100 than the Fp + Rb + Ba configuration, whereas no significant differences were observed for the other selected SCFA-related pathways. Continuous abundance analyses provided no significant evidence for abundance-dependent effects of Rb or Fp or for an Rb × Fp interaction. These findings identify baseline Fp + Rb co-occurrence as a hypothesis-generating microbial configuration associated with responses in selected SCFA-related pathways and provide a basis for independent experimental validation.

## 1 Introduction

Resistant starch (RS) can resist digestion and absorption by human digestive enzymes in the small intestine and instead enters the colon, where it is fermented by the gut microbiota (Dobranowski and Stintzi, 2021). Based on the origin and mechanism of its resistance to digestion, RS is generally classified into five types (RS1–RS5) (Zheng et al., 2026), corresponding to physical entrapment, naturally dense granular structure, retrogradation and recrystallization, chemical modification, and starch–lipid complexes (Sajilata et al., 2006), respectively. In the colon, RS can be fermented by gut microbes to produce short-chain fatty acids (SCFAs), including acetate, propionate, and butyrate (Nogal et al., 2021). These metabolites are not only important energy sources for colonocytes, but also regulate host glucose and lipid metabolism (Kimura et al., 2013; He et al., 2020), intestinal barrier function (Peng et al., 2009; Parada Venegas et al., 2019), and immune and inflammatory (Furusawa et al., 2013; Park et al., 2015) responses through mechanisms such as activation of G protein-coupled receptors and inhibition of histone deacetylases (He et al., 2020). As a result, SCFAs have been implicated in metabolic regulation and the development of metabolic diseases, including diabetes and non-alcoholic fatty liver disease (Canfora et al., 2019; He et al., 2020). In addition, SCFAs, particularly butyrate, have been reported to exert potential antitumor effects by regulating tumor cell proliferation, apoptosis, inflammatory responses and anti-tumor immunity (Blaak et al., 2020; Liu et al., 2018; Zhang et al., 2023). Therefore, promoting SCFA production by the gut microbiota through the intake of resistant starch and other dietary fibers has become a major focus of current research in nutritional intervention and the microbiome.

Among different types of resistant starch, resistant starch type 2 (RS2) naturally occurs in commonly consumed foods such as high-amylose maize starch and has been widely investigated in dietary intervention studies of resistant starch (Bendiks et al., 2020). Due to its compact structure, RS2 is poorly degraded by host digestive enzymes and therefore primarily depends on specific gut microbes capable of initiating resistant starch degradation (Dobranowski and Stintzi, 2021). *Ruminococcus bromii* is considered a key resistant starch degrader in the human gut and has been shown to degrade resistant starch through a cell-surface amylosome complex, in which scaffoldin proteins organize dockerin-containing enzymes, including Amy4, Amy9, Amy10, Amy12, and Amy16, on the bacterial cell surface (Wimmer et al., 2025). Among these enzymes, Amy4 and Amy16 exhibit synergistic activity toward resistant starch, Amy10 and Amy12 participate in debranching starch branch structures, and starch-binding proteins such as Sas6 and Sas20 facilitate bacterial adhesion to insoluble starch granules (Photenhauer et al., 2024; Cerqueira et al., 2022). Meanwhile, *Bifidobacterium adolescentis* also possesses resistant starch-degrading enzymes, including RSD1, RSD2, and RSD3, which contain catalytic domains, carbohydrate-binding modules, and cell wall-anchoring domains (Jung et al., 2020). These upstream degraders have been proposed to release fermentable substrates and intermediate metabolites, such as oligosaccharides, acetate, and lactate, which may contribute to the ecological niche of downstream butyrate-producing bacteria such as *Faecalibacterium prausnitzii* through potential metabolic cross-feeding interactions, Because the abundance and metabolic capacity of these resistant starch-associated taxa vary substantially among individuals, differences in baseline microbiota may contribute to interindividual variability in SCFA-related responses to RS2.

Accumulating evidence indicates that microbial responses to resistant starch vary considerably among individuals and host populations, and that baseline gut microbiota may contribute to this heterogeneity. Previous intervention studies in metabolically distinct populations provide examples of this microbiome-dependent variability. In individuals with obesity, Li et al. (2024) reported that resistant starch intervention was associated with weight loss and improved insulin resistance, with *B. adolescentis* implicated in the microbiota-associated response. Similarly, in patients with non-alcoholic fatty liver disease, Ni et al. (2023) showed that resistant starch intervention reduced intrahepatic triglyceride content and altered the gut microbiota, with microbiota-related effects further supported by fecal microbiota transplantation experiments. These studies demonstrate that resistant starch-induced microbial changes can be linked to metabolic responses in different host contexts, but they also highlight that the taxa and microbial responses identified within individual populations may differ.

More broadly, baseline gut microbial composition and functional capacity have been associated with interindividual variation in microbiota responses to resistant starch supplementation (Devarakonda et al., 2024). However, findings derived from individual intervention cohorts may reflect population-specific host characteristics, baseline diets, disease states, or intervention protocols, making it difficult to distinguish reproducible microbial features from cohort-specific associations. In particular, it remains unclear whether common baseline microbial configurations are consistently associated with SCFA-related functional responses across different populations.

To address this gap, we integrated four publicly available metagenomic intervention datasets representing diverse host backgrounds and performed a cross-cohort analysis of taxonomic and functional responses. By combining cross-cohort species enrichment, SCFA-related pathway responses, baseline microbial stratification, and microbial association analyses, we aimed to identify reproducible baseline microbial configurations associated with changes in selected SCFA-related pathways and to generate hypotheses regarding potential ecological relationships between resistant starch degraders and downstream SCFA-producing bacteria.

## 2 Methods

### 2.1 Data collection and cohort selection

Public metagenomic datasets related to resistant starch intervention were retrieved from the National Center for Biotechnology Information (NCBI) Sequence Read Archive (SRA) and the European Nucleotide Archive (ENA) using the keywords “human gut mcrobiome” and “type 2 resistant starch”. Studies were included if they met the following criteria: (1) the intervention involved RS2; (2) fecal samples were collected from the same participants before and after intervention; (3) whole-genome shotgun metagenomic sequencing data were available; and (4) sufficient metadata were provided to identify sample pairing and intervention status. Studies based only on 16S rRNA gene sequencing, lacking paired pre- and post-intervention samples, or without accessible raw sequencing data were excluded.

For each eligible dataset, sample-level metadata were reviewed to identify baseline and post-intervention or post-exposure samples and to establish the corresponding pairs. Only samples with unambiguous group assignment and complete paired measurements were retained for downstream analysis. Sample-level run accessions, BioSample accessions, BioProject accessions, group assignments, and pairing information are provided in Supplementary Table S2.

### 2.2 Raw metagenomic data processing

Raw sequencing data were downloaded from the NCBI Sequence Read Archive (SRA; https://www.ncbi.nlm.nih.gov/sra). Quality control and adaptor trimming were performed using Trimmomatic (v0.39) with TruSeq adaptor files to remove adaptor sequences and low-quality reads (TruSeq3-PE.fa for paired-end data and TruSeq3-SE.fa for single-end data) (Sewe et al., 2022), and reads shorter than 50 bp were discarded. As these datasets are derived from metagenomic next-generation sequencing, we aligned the quality-filtered reads to the human reference genome (hg19) using Bowtie2 (v2.5.1) to remove host-derived reads (Constantinides et al., 2023). The remaining reads were defined as “clean data” and used for downstream taxonomic and functional profiling. A complete list of included projects, samples, and run accessions is provided in Supplementary Table S2.

### 2.3 Taxonomic and functional profiling

Taxonomic profiling was performed using MetaPhlAn4 (v4.0.3) with default parameters (Blanco-Míguez et al., 2023), and species-level relative abundances were retained for downstream analyses. Functional profiling was performed using HUMAnN3 (v3.6) with default parameters (Beghini et al., 2021) to obtain pathway abundance profiles for each sample. To reduce the influence of low-abundance features on statistical inference and visualization, we filtered taxa and pathways within each cohort following Li et al. (2023) and the Statistical Inference of Associations between Microbial Communities And host phenoTypes (SIAMCAT) guidelines (v1.9.0; https://bioconductor.org/packages/SIAMCAT): taxa and pathways whose maximum relative abundance across all samples in the cohort was <0.001 were removed. Filtered data were then *z*-score standardized for cross-sample comparisons and downstream analyses.

### 2.4 Alpha and beta diversity analyses

To evaluate overall community shifts before and after RS2 intervention, we computed alpha and beta diversity metrics. Alpha and beta diversity analyses were performed separately for each cohort using species-level relative abundance profiles generated by MetaPhlAn4. Alpha diversity was assessed using the Shannon index, which was calculated with the diversity function in the vegan R package. Differences in Shannon diversity between paired before-RS and after-RS samples were evaluated using the paired Wilcoxon signed-rank test (Wen et al., 2023).

Beta diversity was assessed based on Bray–Curtis dissimilarity calculated from species-level relative abundance profiles using the vegdist function in the vegan R package. Principal coordinate analysis (PCoA) was then performed to visualize between-sample community dissimilarities. Differences in overall microbial community composition between before-RS and after-RS samples were tested using permutational multivariate analysis of variance (PERMANOVA) with the adonis2 function in the vegan R package. Statistical significance was assessed with 999 permutations, and *p* values < 0.05 were considered statistically significant (Ferdous et al., 2022).

### 2.5 Biomarker identification using linear discriminant analysis effect size (LEfSe)

We applied LEfSe analysis using the run.lefse function in the microbiomeMarker R package (Cao et al., 2022) to identify taxa and metabolic pathways that differed significantly between pre- and post-RS2 time points. LEfSe outputs an effect size as a linear discriminant analysis (LDA) score, where higher scores indicate stronger between-group differences (Segata et al., 2011). For each cohort, LEfSe was performed on both species-level relative abundance profiles (MetaPhlAn4) and pathway abundance profiles (HUMAnN3). Features with *p* < 0.05 and LDA ≥ 2 were considered candidates. For interpretability, we prefixed LDA scores with “+” or “-” to denote enrichment after RS2 or before RS2, respectively. To capture reproducible cross-population signals, we further required consistent enrichment direction in at least three cohorts for microbial biomarkers and in at least two cohorts for pathway biomarkers. The resulting cross-cohort significant taxa and pathways were used for downstream analyses and visualization (Supplementary Tables S3, S4).

### 2.6 Microbial network analysis

To characterize associations among key taxa and between taxa and SCFA-related pathways, we conducted correlation analyses on baseline samples within each cohort and constructed association networks. Spearman correlations and corresponding significance were computed using the corr.test function in the psych R package (v2.3.9; https://CRAN.R-project.org/package=psych). Associations meeting |*r*| > 0.2 and *p* < 0.05 were retained, and additionally required to show consistent correlation direction (both positive or both negative) in at least two cohorts. Correlation results are summarized in Supplementary Table S5. Network visualization was performed using ggraph (v4.3.2; https://CRAN.R-project.org/package=ggraph). Nodes represent taxa (green) or pathways (yellow), edges represent retained associations, with positive and negative edges shown in blue and red, respectively; edge width can be scaled by the number of supporting cohorts.

### 2.7 Microbial configuration and continuous abundance analyses

To evaluate whether different baseline microbial configurations were associated with SCFA-related pathway responses to RS2 intervention, *Faecalibacterium prausnitzii* (Fp) was considered a downstream butyrate-producing taxon, whereas *Ruminococcus bromii* (Rb) and *Bifidobacterium adolescentis* (Ba) were considered representative upstream resistant starch degraders. Taxon presence at baseline was defined as a non-zero relative abundance. Participants were classified into three mutually exclusive baseline microbial configurations: Fp + Rb (Fp and Rb present, Ba absent), Fp + Ba (Fp and Ba present, Rb absent), and Fp + Rb + Ba (all three taxa present). For each participant, pathway response was quantified as the change in pathway abundance between post-intervention and baseline sample $\;(\Delta Y_{i}=Y_{i,\text{after}}-Y_{i,\text{before}})$ . For each of the five SCFA-related pathways, Fp + Rb was specified as the reference configuration and the following linear regression model was fitted:

$$\begin{array}{l} \Delta Y_{i}=\beta_{0}+\beta_{1}I{\left(\mathrm{Fp}+\mathrm{Ba}\right)}_{i}+\beta_{2}I{\left(\mathrm{Fp}+\mathrm{Rb}+\mathrm{Ba}\right)}_{i}\\ +k=\sum_{k=1}^{K-1}\gamma_{k}I{\left(\text{Cohor}t_{k}\right)}_{i}+\varepsilon_{i}\; \end{array}$$

where I(.) denotes an indicator variable and cohort was included as a categorical covariate to account for systematic differences among intervention studies. Accordingly, $\beta_{1}$ represents the cohort-adjusted difference in pathway response between the Fp + Ba and Fp + Rb configurations, whereas $\beta_{2}$ represents the corresponding difference between the Fp + Rb + Ba and Fp + Rb configurations. The contrast between Fp + Ba and Fp + Rb + Ba was derived from the same fitted model. Because subgroup sizes were unequal and heteroscedasticity could not be excluded, heteroskedasticity-consistent type 3 (HC3) robust standard errors were used for statistical inference. Model-based cohort-adjusted estimated marginal means and 95% confidence intervals were calculated for visualization. Two-sided *p* values <0.05 were considered statistically significant.

Based on the configuration-level analysis, exploratory continuous abundance analyses were further performed within the Fp + Rb configuration to assess whether variation in baseline Rb and Fp abundance was associated with the magnitude of pathway responses. This analysis was restricted to participants in whom both Fp and Rb were detected at baseline and Ba was absent. Baseline relative abundances of Rb and Fp were transformed using log(1+x) and subsequently standardized within each cohort using *Z*-scores to reduce the influence of skewed abundance distributions and between-cohort differences in abundance scale. Continuous abundance analyses were performed for P41-PWY and PWY-5100, which showed the strongest configuration-level signals. For each pathway, the following model was fitted:

$$\begin{array}{l} \Delta Y_{i}=\beta_{0}+\beta_{1}Rb_{z,i}+\beta_{2}Fp_{z,i}+\beta_{3}(Rb_{z,i}\times Fp_{z,i})\\ +\sum_{k=1}^{K-1}\gamma_{k}I{\left(\text{Cohor}t_{k}\right)}_{i}+\varepsilon_{i} \end{array}$$

where $Rb_{z}$ and $Fp_{z}$ denote the log-transformed and within-cohort standardized baseline abundances of Rb and Fp, respectively. The coefficients $\beta_{1}$ and $\beta_{2}$ represent the associations of baseline Rb and Fp abundances with pathway response, respectively, whereas $\beta_{3}$ represents the Rb × Fp interaction term and tests whether the association between the abundance of one taxon and pathway response varies according to the abundance of the other taxon. HC3 robust standard errors were again used for statistical inference, and regression coefficients with 95% confidence intervals were reported.

## 3 Results

### 3.1 Collection and diversity analysis of resistant starch type 2 (RS2) intervention metagenomic cohorts

After screening the publicly available metagenomic datasets according to the predefined inclusion and exclusion criteria, four datasets with paired baseline and post-intervention or post-exposure samples were retained for analysis: PRJNA675102, PRJNA414688, PRJEB15179, and PRJNA703757 (Figure 1A; Supplementary Table S1). These datasets represented distinct experimental or host contexts, including healthy donor-derived *in vitro* resistant starch fermentation (PRJNA675102), overweight/obesity (PRJNA414688), type 2 diabetes (PRJEB15179), and non-alcoholic fatty liver disease (PRJNA703757). In total, the four datasets comprised 30, 54, 148, and 100 samples, respectively, with equal numbers of baseline and post-intervention or post-exposure samples within each dataset.

**Figure 1:**
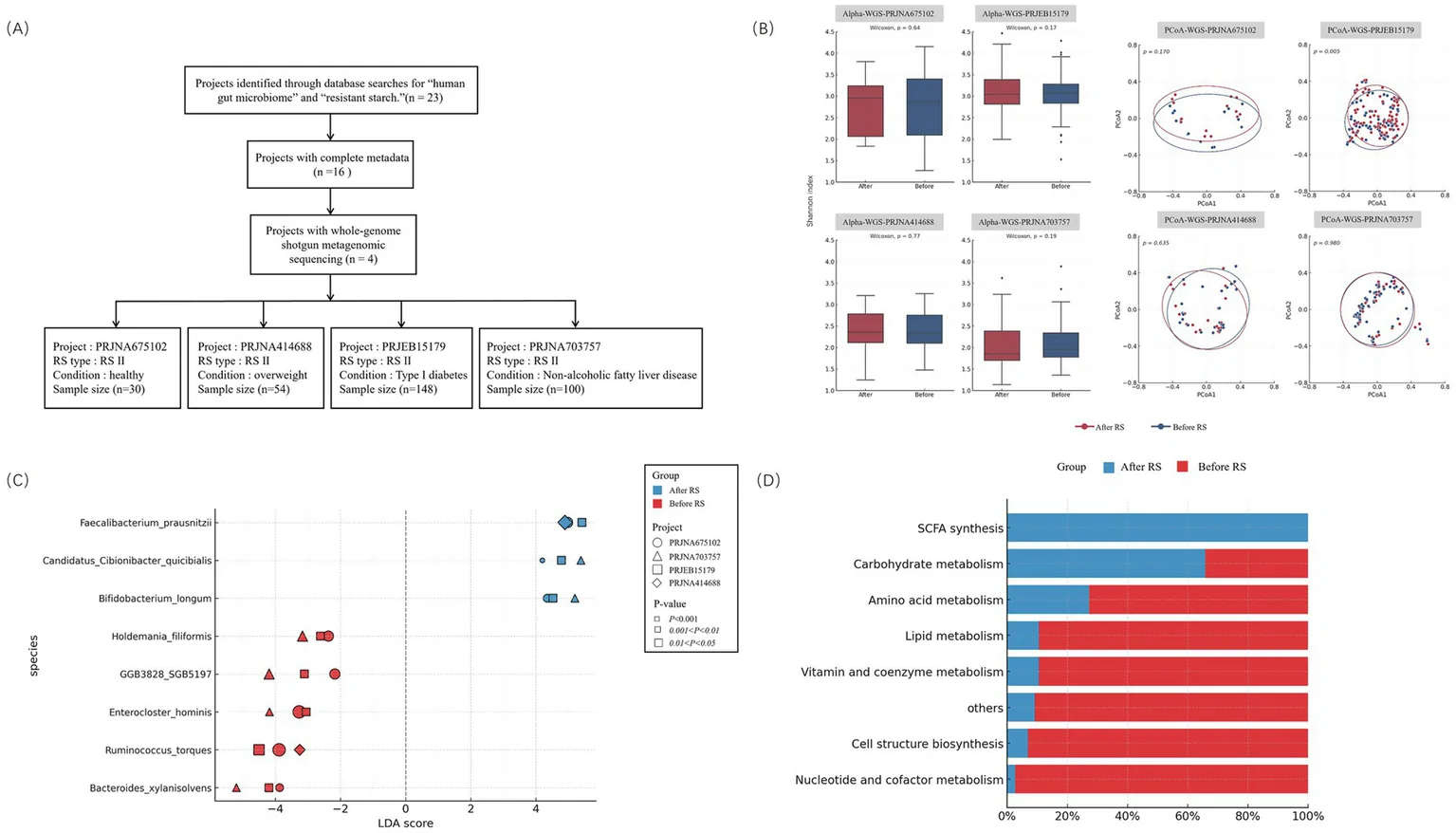
Cross-cohort taxonomic and functional signatures of RS2 intervention. **(A)** Flowchart of cohort collection and included populations. Included cohorts comprised healthy (*n* = 30), obesity (*n* = 54), type 2 diabetes (*n* = 148), and non-alcoholic fatty liver disease (NAFLD) (*n* = 100). *n* denotes total samples; pre/post sample sizes are equal due to paired design. **(B)** Alpha and beta diversity before (red) and after resistant starch type 2 (RS2) (blue). Left: Shannon index compared by paired Wilcoxon test. Right: PCoA based on Bray–Curtis dissimilarity; group separation assessed by PERMANOVA. Each point represents a sample. **(C)** Differential taxa that show consistent alteration directions across cohorts. Differential taxa were identified by LEfSe with LDA ≥ 2 and *p* < 0.05 within cohorts. Taxa with consistent direction in ≥3 cohorts were defined as cross-cohort biomarkers and retained for visualization. Color indicates enrichment direction (red, pre; blue, post). Shape indicates cohort. Bubble size reflects *p*-value (smaller bubbles indicate smaller *p*-values). **(D)** Bar plot summarizing broad functional categories of cross-cohort differential pathways. LEfSe was performed on HUMAnN pathway abundances, and pathways with LDA ≥ 2, *p* < 0.05, and consistent enrichment direction in ≥2 cohorts were retained. Detailed daughter categories for each pathway are provided in Supplementary Table S4. Pathways were collapsed into broad functional categories for visualization, and the number in parentheses after each broad category label indicates the number of daughter categories included. Red and blue indicate before-RS- and after-RS-enriched pathways, respectively. (PCoA, principal coordinate analysis; PERMANOVA, permutational multivariate analysis of variance; LEfSe, linear discriminant analysis effect size; LDA, linear discriminant analysis; NAFLD, non-alcoholic fatty liver disease; RS2, resistant starch type 2).

We first evaluated whether RS2 intervention altered overall gut microbial diversity. Alpha diversity based on the Shannon index showed no significant difference between before-RS and after-RS samples in any of the four cohorts (Figure 1B). Beta diversity analysis based on Bray–Curtis dissimilarity showed that only the PRJEB15179 cohort exhibited a significant difference in overall community structure before and after intervention, whereas the other three cohorts showed no obvious separation (Figure 1B). These results indicate that RS2 intervention did not induce consistent global shifts in gut microbial diversity across cohorts, suggesting that its effects may be more evident at the level of specific taxa and metabolic pathways.

### 3.2 Cross-cohort taxonomic and functional signatures of RS2 intervention

To identify microbial biomarkers showing consistent responses across cohorts, we performed LEfSe analysis separately for each of the four cohorts. A total of 333 species showed significant abundance changes in at least one cohort. We first extracted the top 20 species ranked by absolute LDA score in each cohort for comparison (Supplementary Figure S1). The top-ranked species were not fully consistent across cohorts, indicating cohort-specific differences in taxonomic responses after RS2 intervention.

Because individual species did not always show the same enrichment direction across cohorts, we further screened species with consistent enrichment directions in three or more cohorts. This analysis identified eight cross-cohort consistent species, including five before-RS enriched species and three after-RS enriched species (Figure 1C; Supplementary Table S3). The three after-RS enriched species were *F. prausnitzii*, *Bifidobacterium longum*, and *Candidatus Cibionibacter quicibialis*.

To further characterize functional responses after RS2 intervention, we performed LEfSe analysis on metabolic pathway abundance profiles. Pathways showing consistent enrichment directions in two or more cohorts were retained and classified according to their biological functions. The proportions of before-RS and after-RS enriched pathways were then calculated within each functional category (Figure 1D; Supplementary Figure S2). Because *F. prausnitzii* is a known butyrate-producing bacterium, the butyrate synthesis pathways PWY-5676 and CENTFERM-PWY, which were enriched in one cohort, were also included in subsequent analyses (Supplementary Table S4). Among the classified pathways, short-chain fatty acid synthesis and carbohydrate metabolism showed higher proportions of after-RS enriched pathways. Within the short-chain fatty acid synthesis category, the enriched pathways were mainly related to acetate and butyrate production.

### 3.3 Baseline *F. prausnitzii* is associated with stronger SCFA-related pathway responses

Based on the cross-cohort microbial markers and functional pathway signatures described above, we constructed a metabolic framework of the RS2 response by integrating pathways enriched in the SCFA synthesis and carbohydrate metabolism categories (Jiang et al., 2025) (Figure 2A). This framework summarizes the potential metabolic route from resistant starch degradation to the generation of pyruvate and acetyl coenzyme A (acetyl-CoA), followed by acetate/lactate production and downstream butyrate synthesis. The framework also highlights the potential metabolic connection between acetate production and butyrate synthesis (Louis and Flint, 2009).

**Figure 2:**
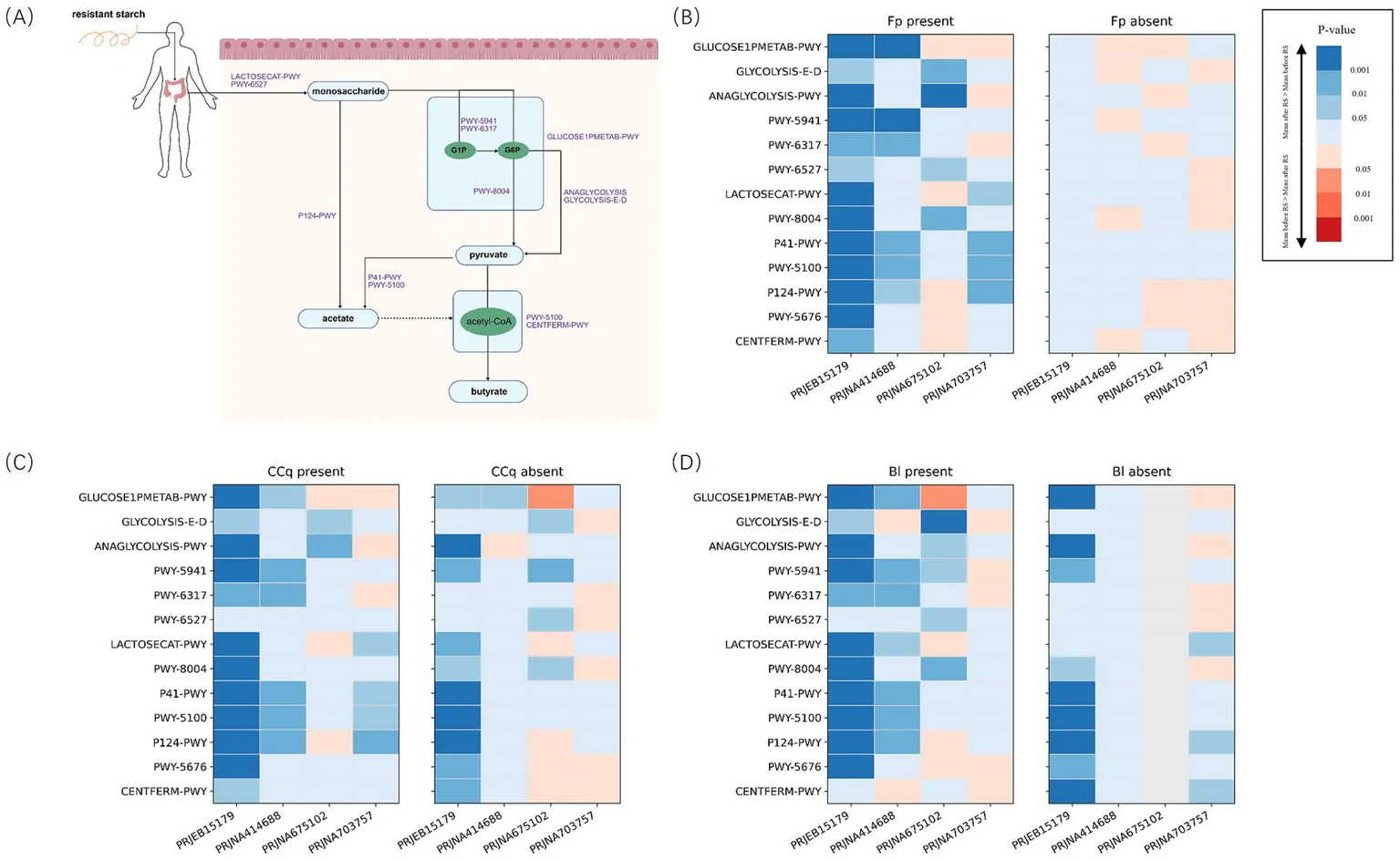
Baseline *F. prausnitzii* is associated with enhanced SCFA-related pathway responses after RS2 intervention. **(A)** Schematic representation of the metabolic framework linking resistant starch utilization to short-chain fatty acid (SCFA) production. Resistant starch-derived carbohydrates enter central carbohydrate metabolism and are converted through intermediates including glucose-1-phosphate (G1P), glucose-6-phosphate (G6P), pyruvate, and acetyl-CoA, ultimately contributing to acetate and butyrate production. Purple labels indicate the corresponding MetaCyc pathways included in the analysis, and arrows indicate the proposed metabolic connections among substrates, intermediates, and SCFA end products. **(B–D)** Heatmaps showing pre–post changes in selected carbohydrate metabolism- and SCFA-related pathways after stratification according to the baseline presence or absence of **(B)** *Faecalibacterium prausnitzii* (Fp), **(C)** *Candidatus Cibionibacter quicibialis (CCq)*, and **(D)** *Bifidobacterium longum* (Bl). A species was considered present when it was detected in the corresponding baseline sample. For Fp, the numbers of baseline samples in the present/absent groups were 72/2 in PRJEB15179, 24/3 in PRJNA414688, 13/2 in PRJNA675102, and 44/6 in PRJNA703757. For CCq, the corresponding numbers were 51/23, 18/9, 8/7, and 33/17, respectively; for Bl, they were 50/24, 19/8, 12/3, and 37/13, respectively. Rows represent metabolic pathways and columns represent the four cohorts (PRJEB15179, PRJNA414688, PRJNA675102, and PRJNA703757). Within each baseline stratum and cohort, pathway abundances before and after intervention were compared. Blue indicates a higher mean pathway abundance after intervention than before intervention, whereas red indicates a higher mean abundance before intervention than after intervention. Color intensity represents the *p* value of the pre–post comparison, with darker colors indicating smaller *p* values and stronger statistical evidence for a difference. (SCFA, short-chain fatty acid; RS2, resistant starch type 2; Fp, *F. prausnitzii*; CCq, Candidatus Cibionibacter quicibialis; Bl, *B. longum*).

We next examined whether the baseline presence of after-RS enriched species was associated with changes in these metabolic pathways. Three species that were consistently enriched after RS2 intervention in Figure 1C, namely *F. prausnitzii* (Fp), *B.longum*, and *Candidatus Cibionibacter quicibialis*, were selected for baseline presence/absence analysis. For each species, samples were stratified according to whether the species was detected in the baseline microbiota, and changes in SCFA synthesis and carbohydrate metabolism pathways before and after RS2 intervention were compared (Figure 2B).

In the baseline presence/absence stratification, group sizes varied substantially among species and cohorts. *F. prausnitzii* was detected in most baseline samples, with present/absent sample numbers of 72/2 in PRJEB15179, 24/3 in PRJNA414688, 13/2 in PRJNA675102, and 44/6 in PRJNA703757. In comparison, the presence/absence groups were more balanced for Candidatus Cibionibacter quicibialis (51/23, 18/9, 8/7, and 33/17, respectively) and *B. longum* (50/24, 19/8, 12/3, and 37/13, respectively) (Figures 2B–D).

Descriptively, the *F. prausnitzii*-present strata showed a greater number of pathways with higher post-intervention abundance and stronger statistical evidence for pre–post differences than the corresponding *F. prausnitzii*-absent strata (Figure 2B). However, because *F. prausnitzii* was highly prevalent at baseline and the absent groups contained very few samples in several cohorts, these presence/absence comparisons should be interpreted cautiously. The corresponding stratifications for C. Cibionibacter quicibialis and *B. longum* showed more heterogeneous patterns across cohorts (Figures 2C,D). Thus, this analysis was considered exploratory and was used to motivate subsequent analyses of the relationships among *F. prausnitzii*, resistant starch degraders, and SCFA-related pathways rather than to establish baseline presence of *F. prausnitzii* as an independent predictor of pathway response.

### 3.4 Baseline abundance of primary RS degraders correlate with SCFA-related pathways

Because *F. prausnitzii* is not considered a primary resistant starch degrader (Dobranowski and Stintzi, 2021), we next examined whether its response to RS2 intervention was associated with the presence of upstream resistant starch-degrading bacteria at baseline. *R. bromii* (Wimmer et al., 2025)and *B. adolescentis* (Klostermann et al., 2025) were selected as representative resistant starch degraders. We compared the responses of the three after-RS enriched species according to whether either *R. bromii* or *B. adolescentis* was present at baseline. The numbers of samples in the “Rb or Ba present”/“Rb and Ba both absent” groups were 11/4 in PRJNA675102, 58/16 in PRJEB15179, 18/9 in PRJNA414688, and 35/15 in PRJNA703757. Among these species, *F. prausnitzii* showed the clearest descriptive difference according to baseline Rb/Ba presence: its after-RS enrichment was more evident when either degrader was present, whereas this response was attenuated when both degraders were absent (Figure 3A).

**Figure 3:**
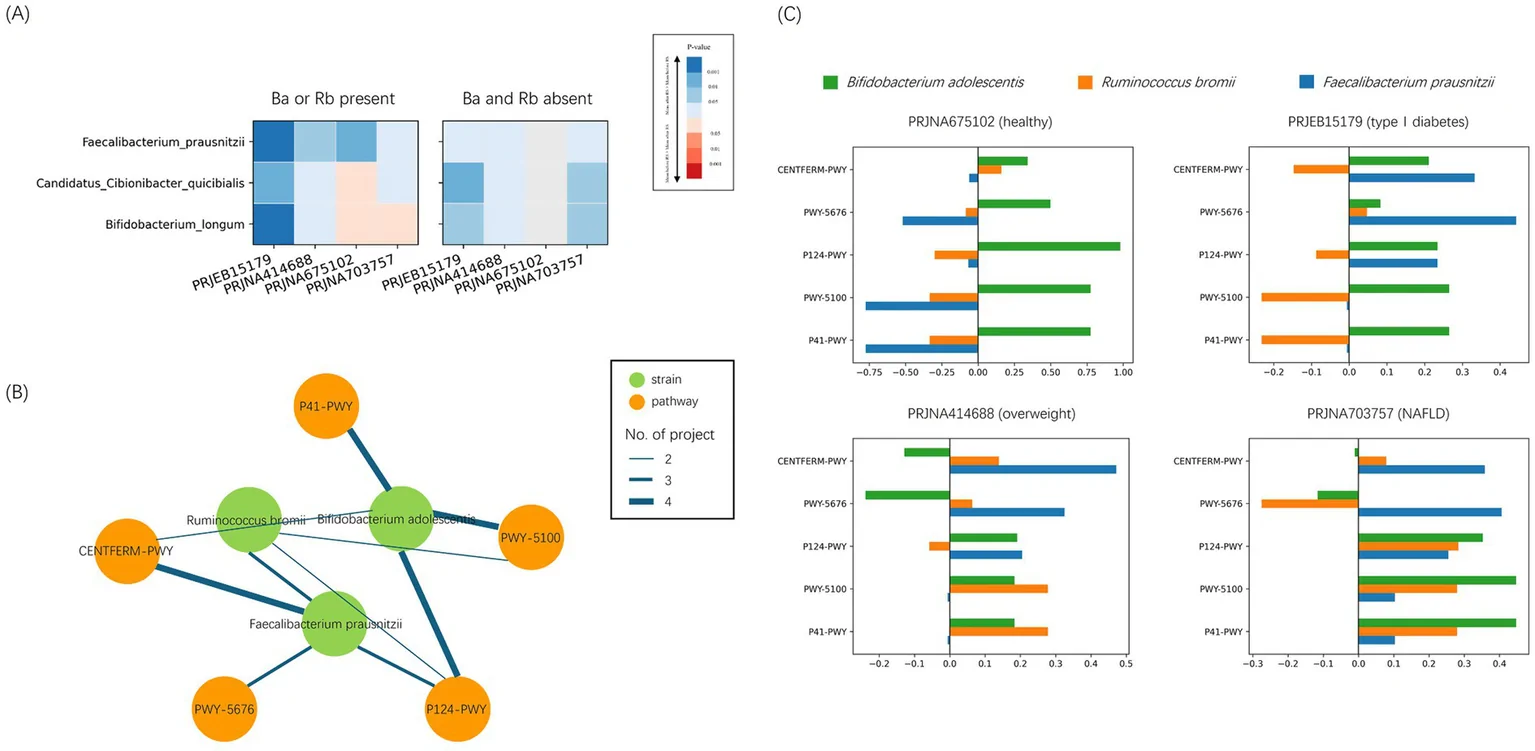
Baseline abundance of primary resistant starch degraders is associated with SCFA-related pathways. **(A)** Heatmaps comparing the pre–post abundance changes of three taxa consistently enriched after intervention—*Faecalibacterium prausnitzii* (Fp), *Candidatus Cibionibacter quicibialis* (CCq), and *Bifidobacterium longum* (Bl)—according to the baseline presence of the representative resistant starch degraders *Bifidobacterium adolescentis* (Ba) and *Ruminococcus bromii* (Rb). Samples were classified as “Ba or Rb present” when at least one of the two species was detected at baseline and as “Ba or Rb absent” when neither species was detected. The numbers of baseline samples in the “Ba or Rb present”/“Ba and Rb both absent” groups were 11/4 in PRJNA675102, 58/16 in PRJEB15179, 18/9 in PRJNA414688, and 35/15 in PRJNA703757. Columns represent the four cohorts. Blue indicates a higher mean taxon abundance after intervention than before intervention, whereas red indicates a higher mean abundance before intervention than after intervention. Color intensity represents the *p* value of the pre–post comparison, with darker colors indicating smaller *p* values. **(B)** Cross-cohort association network among three key bacterial taxa (Ba, Rb, and Fp) and five SCFA-related pathways. Spearman correlations were calculated separately using baseline samples within each cohort. Associations with |*r*| > 0.2 and *p* < 0.05 were retained and were further required to show a consistent direction in at least two cohorts. For taxon–pathway associations, cohort-level *p* values were additionally combined using Fisher’s method. Green nodes represent bacterial taxa and orange nodes represent metabolic pathways. Edges indicate retained associations, and edge width represents the number of cohorts supporting the association (2–4 cohorts). The five pathways comprise the acetate/lactate-related pathways P41-PWY, PWY-5100, and P124-PWY and the butyrate-related pathways PWY-5676 and CENTFERM-PWY. **(C)** Cohort-specific Spearman correlations between Ba, Rb, and Fp and the five SCFA-related pathways. Results are shown separately for PRJNA675102 (healthy), PRJEB15179 (type 2 diabetes), PRJNA414688 (overweight/obesity), and PRJNA703757 (non-alcoholic fatty liver disease, NAFLD). The *x*-axis represents the Spearman correlation coefficient (*r*), with positive and negative values indicating positive and inverse associations, respectively. Green, orange, and blue bars represent Ba, Rb, and Fp, respectively.

We then investigated baseline microbial associations among *R. bromii*, *B. adolescentis*, and *F. prausnitzii*, as well as their associations with five SCFA-related pathways. Spearman correlations were calculated separately within each cohort, and associations showing consistent directions in at least two cohorts were retained. For taxon-pathway associations, cohort-level correlation results were further integrated using Fisher’s method (Figure 3B). At the taxon-taxon level, only *R. bromii* and *F. prausnitzii* showed a consistent positive association across cohorts. At the taxon-pathway level, *B. adolescentis* was positively associated with acetate-related pathways, including P41-PWY, PWY-5100, and P124-PWY, whereas *R. bromii* was mainly positively associated with PWY-5100 and P124-PWY. In contrast, *F. prausnitzii* was positively associated with butyrate-related pathways, including CENTFERM-PWY and PWY-5676, and was also positively associated with the acetate-related pathway P124-PWY (Figure 3B). These results indicate that upstream resistant starch degraders and downstream SCFA-producing bacteria exhibited distinct but potentially connected pathway association patterns.

To further evaluate cohort-specific variation, we separately visualized the correlations between the three key bacteria and the five SCFA-related pathways in each cohort (Figure 3C). The direction and strength of these associations varied across populations. For example, in the PRJNA675102 healthy cohort, *B. adolescentis* was positively correlated with all five SCFA-related pathways, whereas in the PRJNA414688 overweight/obese cohort, its correlations with butyrate-related pathways were negative. These findings suggest that the relationships between resistant starch degraders and SCFA-related pathways are partly population-dependent, supporting the need for cross-cohort comparison to distinguish reproducible associations from cohort-specific patterns.

### 3.5 Baseline Fp + Rb microbial configuration is associated with stronger responses in selected SCFA-related pathways

To further evaluate the associations between specific baseline microbial configurations and SCFA-related pathway responses, we compared three mutually exclusive baseline configurations comprising Fp + Rb, Fp + Ba, and Fp + Rb + Ba using cohort-adjusted linear regression models (Figure 4A).

**Figure 4:**
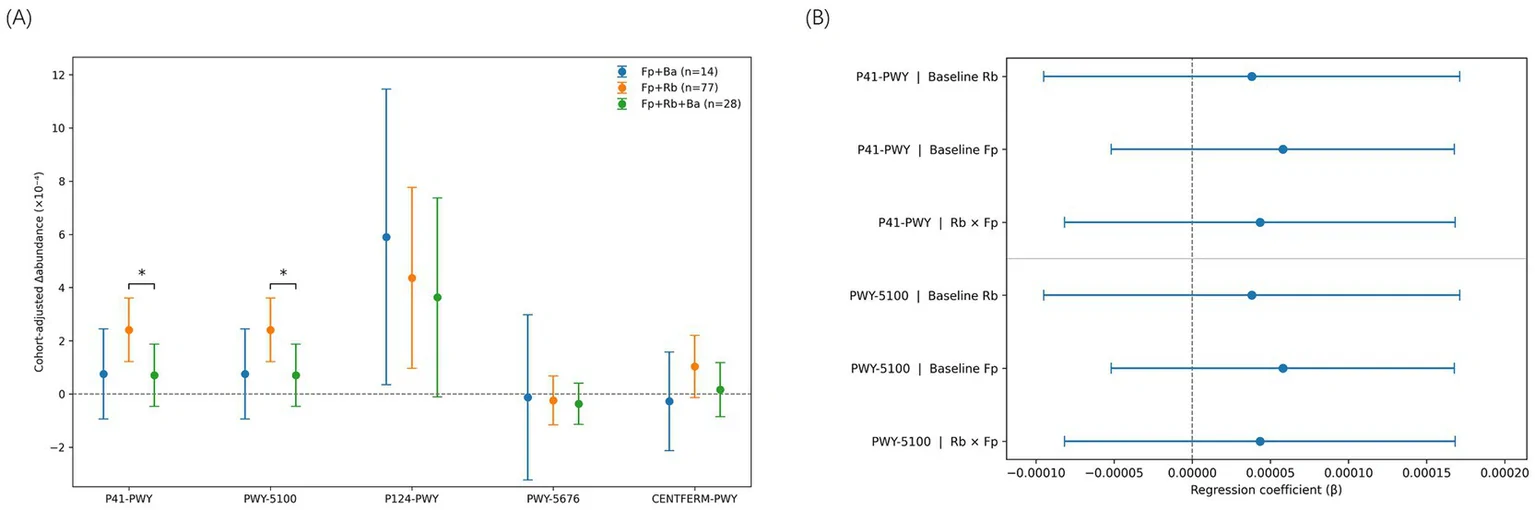
Baseline Fp + Rb microbial configuration is associated with stronger responses in selected SCFA-related pathways. **(A)** Cohort-adjusted changes in the abundances of five SCFA-related pathways across three mutually exclusive baseline microbial configurations: Fp + Ba (*n* = 14), Fp + Rb (*n* = 77), and Fp + Rb + Ba (*n* = 28). Pathway response was defined as Δabundance = After—Before. Points represent cohort-adjusted estimated marginal means and error bars indicate 95% confidence intervals derived from linear regression models with HC3 robust standard errors. Asterisks indicate significant pairwise contrasts between microbial configurations based on two-sided *p* < 0.05. The dashed horizontal line indicates no change (Δabundance = 0). **(B)** Continuous abundance analysis within the Fp + Rb baseline configuration (Fp and Rb present and Ba absent; n = 77). Baseline abundances of Rb and Fp were log-transformed and standardized within each cohort before model fitting. Points represent regression coefficients (*β*) for baseline Rb abundance, baseline Fp abundance, and the Rb × Fp interaction, and horizontal error bars represent 95% confidence intervals from cohort-adjusted linear regression models with HC3 robust standard errors. The dashed vertical line indicates *β* = 0. (Fp, *Faecalibacterium prausnitzii*; Rb, *Ruminococcus bromii*; Ba, *Bifidobacterium adolescentis*; SCFA, short-chain fatty acid; HC3, heteroskedasticity-consistent type 3).

The associations between microbial configuration and pathway response were pathway-specific. For the acetate/lactate-related pathways P41-PWY and PWY-5100, the cohort-adjusted increases were significantly greater in the Fp + Rb group than in the Fp + Rb + Ba group. In contrast, no significant differences among the three microbial configurations were observed for P124-PWY, PWY-5676, or CENTFERM-PWY. Although the adjusted increase in CENTFERM-PWY was numerically greater in the Fp + Rb group, its 95% confidence interval crossed zero. These findings indicate that the association between the baseline Fp + Rb configuration and RS2-related functional responses was pathway-specific, with the strongest signals observed for P41-PWY and PWY-5100 rather than a consistent enhancement across all SCFA-related pathways.

We subsequently examined whether interindividual variation in the baseline abundances of Rb and Fp within the Fp + Rb configuration was associated with the magnitude of the responses in P41-PWY and PWY-5100 (Figure 4B). This analysis was restricted to participants in whom both Fp and Rb were detected at baseline and Ba was absent (*n* = 77). In continuous abundance models adjusted for cohort effects, the regression coefficients for baseline Rb abundance, baseline Fp abundance, and the Rb × Fp interaction were all positive for P41-PWY, with *β* = 3.80 × 10^−5^ (*p* = 0.576), *β* = 5.80 × 10^−5^ (*p* = 0.301), and *β* = 4.33 × 10^−5^ (*p* = 0.498), respectively. PWY-5100 yielded the same model estimates. However, the corresponding 95% confidence intervals all crossed zero, indicating no statistically significant abundance-dependent associations or Rb × Fp interaction. Overall, these results more strongly support an association between the baseline Fp + Rb microbial configuration itself and responses in selected pathways, while providing insufficient evidence that higher baseline abundances of Rb and Fp within this configuration are associated with stronger pathway responses.

## 4 Discussion

In this study, we integrated four publicly available metagenomic datasets representing diverse experimental and host contexts to identify reproducible microbial and functional signals associated with resistant starch-related intervention or exposure. Although no consistent changes in alpha diversity were observed across datasets and significant beta-diversity alteration was detected in only one dataset, cross-cohort analyses identified more reproducible responses at the levels of specific taxa and metabolic pathways. *F. prausnitzii* was among the taxa consistently enriched after intervention or exposure across multiple datasets. In addition, among the cross-cohort differential pathways retained in our analysis, SCFA synthesis and carbohydrate metabolism showed higher proportions of post-intervention/post-exposure enrichment. These findings suggest that resistant starch-associated microbiome responses may be more readily detected at the level of specific microbial taxa and functional pathways than through global community diversity measures.

Exploratory baseline presence/absence stratification further suggested that samples in which *F. prausnitzii* was detected at baseline tended to show a greater number of post-intervention increases in selected SCFA- and carbohydrate metabolism-related pathways than samples in which it was not detected. However, *F. prausnitzii* was highly prevalent at baseline, resulting in very small absent groups in several datasets. Therefore, these comparisons should not be interpreted as establishing baseline *F. prausnitzii* as an independent predictor of pathway response. Rather, they provided a hypothesis-generating basis for examining its ecological relationships with representative resistant starch degraders. Because *F. prausnitzii* is an important butyrate-producing bacterium but is not generally considered a primary resistant starch degrader (Louis and Flint, 2009; Kim et al., 2024), we further examined *R. bromii* and *B. adolescentis* as representative upstream degraders (Jung et al., 2020; Wimmer et al., 2025; Klostermann et al., 2025). The post-intervention enrichment of *F. prausnitzii* was descriptively more evident when at least one of these degraders was detected at baseline than when both were absent. Given the unequal subgroup sizes and observational nature of this analysis, this pattern should be interpreted as consistent with a potential ecological relationship rather than evidence of microbial dependence or causal cross-feeding (Dobranowski and Stintzi, 2021).

The baseline association analysis provided additional support for this hypothesis. At the taxon–taxon level, *R. bromii* and *F. prausnitzii* were the only pair showing a consistent positive association across multiple datasets. At the taxon–pathway level, *R. bromii* was mainly associated with acetate-related pathways, whereas *F. prausnitzii* was associated with both acetate-related and butyrate-related pathways. These patterns are consistent with potentially complementary ecological roles of resistant starch degradation and downstream SCFA-related metabolism (Dobranowski and Stintzi, 2021; Louis and Flint, 2009; Wimmer et al., 2025), although correlation alone cannot establish metabolic cross-feeding. In contrast, the associations of *B. adolescentis* with SCFA-related pathways were more context-dependent. For example, *B. adolescentis* was positively associated with the five selected SCFA-related pathways in the healthy donor-derived *in vitro* fermentation dataset, whereas its associations with butyrate-related pathways differed in the overweight/obesity dataset. This heterogeneity further highlights the importance of cross-dataset analysis for distinguishing reproducible associations from context-specific patterns.

The revised microbial-configuration analysis provided a more focused evaluation of these associations. When samples were assigned to mutually exclusive baseline Fp + Rb, Fp + Ba, and Fp + Rb + Ba configurations, the Fp + Rb configuration was associated with significantly greater cohort-adjusted increases in the acetate/lactate-related pathways P41-PWY and PWY-5100 than the Fp + Rb + Ba configuration. In contrast, no significant differences among configurations were observed for P124-PWY, PWY-5676, or CENTFERM-PWY. Thus, the association with the Fp + Rb configuration was pathway-specific rather than indicative of a uniformly stronger SCFA-related response, consistent with previous observations that microbial and functional responses to resistant starch can vary across taxa, pathways, and host contexts (Bendiks et al., 2020; Devarakonda et al., 2024). More broadly, recent evidence highlights that SCFA-related responses to dietary substrates are shaped by the interplay among gut microbiota composition, dietary factors, and host characteristics, supporting the potential importance of baseline microbial configurations in determining interindividual functional responses (Sankarganesh et al., 2025) Furthermore, continuous abundance analyses within the Fp + Rb/Ba− subgroup did not identify significant associations of baseline Rb abundance, baseline Fp abundance, or the Rb × Fp interaction with pathway responses. These findings therefore provide greater support for an association with the baseline microbial configuration itself than for an abundance-dependent or synergistic effect of Rb and Fp. This interpretation is biologically plausible given the established role of *R. bromii* in resistant starch degradation and the butyrate-producing capacity of *F. prausnitzii*, although these complementary functions do not by themselves demonstrate synergistic interaction (Louis and Flint, 2009; Dobranowski and Stintzi, 2021; Wimmer et al., 2025).

Several limitations should be considered. First, all analyses were based on publicly available metagenomic datasets, and the same datasets were used for the exploratory identification and subsequent evaluation of candidate microbial features; therefore, the findings do not constitute independent validation and cannot establish causality (Walter et al., 2020). Second, the included datasets differed in experimental design, host background, disease status, dietary context, and intervention or exposure protocols, which may have contributed to between-dataset heterogeneity (Bendiks et al., 2020; Devarakonda et al., 2024). Third, some baseline presence/absence strata were small and unbalanced, particularly for *F. prausnitzii*, limiting the robustness of these exploratory comparisons (Ferdous et al., 2022). Fourth, the analyses of Fp, Rb, and Ba were hypothesis-driven and targeted rather than microbiome-wide screening of all possible baseline predictors. Finally, metagenomic pathway abundance reflects predicted functional potential rather than actual metabolic activity or SCFA concentrations (Beghini et al., 2021). Future studies using independent and larger cohorts, more balanced subgroup designs, taxon-resolved functional profiling, targeted metabolomics, and controlled co-culture or gnotobiotic experiments will be required to validate these associations and clarify their underlying mechanisms (Walter et al., 2020).

Overall, this cross-dataset analysis identified the baseline co-occurrence of *F. prausnitzii* and *R. bromii* as being associated with greater increases in selected SCFA-related pathways, particularly P41-PWY and PWY-5100, following resistant starch-related intervention or exposure. However, the continuous abundance analysis did not support a significant abundance-dependent Rb × Fp interaction. These findings should therefore be considered hypothesis-generating evidence for a potentially relevant baseline microbial configuration rather than evidence of an optimal or synergistic microbial combination, and they provide a basis for further experimental and independently replicated investigation.

## Data Availability

The original contributions presented in the study are included in the article/Supplementary material, further inquiries can be directed to the corresponding author/s.
