# Supplementary material for: Baseline co-occurrence of Faecalibacterium prausnitzii and Ruminococcus bromii is associated with greater increases in selected short-chain fatty acid-related pathways following resistant starch type 2 intervention across diverse populations
Source: Front Microbiol. 2026 Oct 7;17:1959463. doi: 10.3389/fmicb.2026.1959463 (PMC13682993; doi:10.3389/fmicb.2026.1959463)
Supplement: Supplementary file 1 [file supplementary_file_1.docx]

Figure S1 LEfSe analysis was performed separately for the PRJNA675102, PRJEB15179, PRJNA414688, and PRJNA703757 cohorts, and the top 20 differential species ranked by absolute LDA score in each cohort are shown. Blue bars indicate species enriched after RS2 intervention (case), whereas red bars indicate species enriched before intervention (control). The x-axis represents the LDA score, and the y-axis shows the species names.

Figure S2 After performing LEfSe analysis and functional classification of differential metabolic pathways across the four RS2 intervention cohorts, we found that the proportion of post-intervention enriched pathways was relatively high in the carbohydrate metabolism and SCFA synthesis categories. Therefore, representative pathways enriched after RS2 intervention within these two categories are further displayed in the bubble plot. The x-axis represents the LDA score, and the y-axis shows the pathway names. Blue indicates pathways enriched after RS2 intervention, whereas red indicates pathways enriched before intervention. Different bubble shapes represent different cohorts, and bubble size indicates the level of significance.
